# Crowned Dens Syndrome Presenting as Failure to Thrive in a Patient With Moderate Cognitive Impairment

**DOI:** 10.7759/cureus.78158

**Published:** 2025-01-28

**Authors:** Gustavo Castellanos, Gregory L Stone, Amir A Razmjou

**Affiliations:** 1 Department of Medicine, UCLA Medical Center/David Geffen School of Medicine, Los Angeles, USA; 2 Division of Pulmonary, Critical Care, and Sleep Medicine, Department of Medicine, UCLA Medical Center/David Geffen School of Medicine, Los Angeles, USA; 3 Division of Rheumatology, Department of Medicine, UCLA Medical Center/David Geffen School of Medicine, Los Angeles, USA; 4 Division of Rheumatology, Department of Medicine, Greater Los Angeles VA Healthcare System, Los Angeles, USA

**Keywords:** acute neck pain and stiffness, calcium pyrophosphate deposition disease (cppd), cervical pain, crowned dens syndrome, esr (erythrocyte sedimentation rate), recurrent fever, steroid taper, transverse ligament

## Abstract

Crowned dens syndrome (CDS) is a rare condition characterized by the deposition of calcium pyrophosphate (CPP) or hydroxyapatite crystals around the odontoid process of the C2 vertebra, often presenting with acute neck pain, reduced cervical mobility, and systemic inflammatory response. This case describes a 68-year-old male patient with a history of type 2 diabetes, hypertension, and benign prostatic hyperplasia who presented with generalized weakness and failure to thrive. This atypical presentation initially masked symptoms of CDS, delaying the diagnosis until the patient developed acute neck pain and stiffness, prompting further imaging. CT imaging revealed calcifications around the odontoid process, confirming the diagnosis of CDS. Given the severity of the symptoms, corticosteroids were administered, resulting in significant clinical improvement. This case highlights the importance of considering CDS in the differential diagnosis of older patients presenting with systemic inflammatory symptoms and unexplained neck pain, particularly when initial workups are inconclusive.

## Introduction

Crowned dens syndrome (CDS) involves the deposition of calcium pyrophosphate (CPP) [1] or hydroxyapatite crystals on the cruciate ligament overlying the dens of the C2 vertebra [2], leading to an inflammatory response that manifests as neck pain and systemic inflammatory symptoms. CDS is primarily associated with advanced age and typically presents with neck pain, fever, and elevated inflammatory markers, such as C-reactive protein (CRP), erythrocyte sedimentation rate (ESR), and leukocytosis [3]. This clinical presentation can mimic that of meningitis (fever, meningismus, elevated inflammatory markers) or other conditions such as polymyalgia rheumatica (PMR) (neck/shoulder pain, fever, malaise) [4]. When evaluating a patient with neck pain, fever, and elevated inflammatory markers, a high index of suspicion for CDS can help prevent diagnostic delays and guide a more targeted workup. This report presents a case of CDS with a delayed diagnosis.

## Case presentation

A 68-year-old male patient with a past medical history of chronic type 2 diabetes, hypertension, hyperlipidemia, and benign prostatic hyperplasia (the exact duration of chronic conditions was unclear due to long-standing disease) presented to the emergency department of an urban public academic medical center in Southern California with subacute progressive generalized weakness. The patient was brought in by first responders after being found on the ground, unable to stand, during a wellness check. Two weeks prior, he had traveled to Central America, where he was similarly found on the ground by family members after being out of contact for several days. He was taken to a local hospital, diagnosed with a transient ischemic attack, and discharged. Upon his return to the United States, the patient became confused and disoriented, prompting emergency intervention by family members and emergency services.

Upon presentation to the emergency department, the patient was confused and disoriented. He reported back pain but denied fever, chills, or headache. Due to his confusion, an extensive history could not be obtained. Given the patient’s weakness, altered mentation, and recent trauma, imaging studies were promptly performed. CT of the head showed no acute intracranial process, while MRI of the brain revealed generalized atrophy, nonspecific white matter changes, and prominent right frontal and temporal lobe atrophy, without evidence of acute stroke or vascular occlusions. MRI of the lumbar spine revealed mild degenerative changes at L5-S1. Upon admission, the patient's vital signs were stable, and he was afebrile. Physical examination revealed mild knee pain and left-sided weakness, with gait abnormalities disproportionate to the weakness observed on the neurologic examination. Laboratory studies taken on arrival (Table 1) revealed a white blood cell count of 9.3 K/uL on CBC, along with elevated C-reactive protein (3.61 mg/dL), ESR (85 mm/hr), and creatine kinase (395 U/L). The patient’s renal function remained normal and stable throughout the hospitalization. Hepatic function was unremarkable, with liver enzymes and bilirubin within normal limits. Urinalysis showed elevated leukocyte esterase (250 Leu/uL), white blood cells (24/HPF), red blood cells (10/HPF), and granular casts (7/LPF). Urine culture and toxicology screen were negative. HIV, rapid plasma reagin (RPR), and QuantiFERON Gold tests were all within normal limits. 

**Table 1:** Patient's significant lab results "H" indicates that the corresponding result is higher than the reference range.

Test	Result	Flag	Reference range
Complete blood count (CBC)			
Erythrocyte sedimentation rate (ESR)	85 mm/h	H	0-20
White blood cell (WBC) count	9.3 K/uL		4.5-11
Comprehensive metabolic panel (CMP)			
C-reactive protein (CRP)	3.61 mg/dL	H	≤0.744
Creatine kinase (CK)	395 U/L	H	40-280
Urinalysis (UA)			
Leukocyte esterase	250 Leu/uL	H	−25
WBC in urine	24/HPF	H	0-5
Red blood cells (RBCs in urine)	10/HPF	H	0-5
Granular casts	7/LPF	H	None
Other			
HIV	Negative		Negative
Rapid plasma reagin (RPR)	Non-reactive		Non-reactive
QuantiFERON Gold	Negative		Negative

The patient subsequently developed a fever, and abnormal urinalysis findings suggestive of a UTI were noted (high leukocyte esterase, WBCs and RBCs in urine). Urine cultures were obtained, and a CT of the pelvis was ordered, revealing a possible fluid collection around an enlarged prostate. Although initially suspected to be prostatitis by the primary team, urology and infectious disease consultants determined it was unlikely to be related to the patient's presenting syndrome.

On day six of hospitalization, the patient developed recurrent fevers accompanied by neck pain and stiffness, raising concern for possible meningitis, given his recent travel and confusion. A lumbar puncture was performed, and cerebrospinal fluid analysis was unremarkable, showing no pleocytosis, normal glucose, and mildly elevated protein of 64 mg/dL, with extensive negative bacterial, viral, and fungal PCR and culture studies. Despite empirical treatment with antimicrobials, including acyclovir, vancomycin, piperacillin-tazobactam, and ceftriaxone, the patient showed no clinical improvement. 

**Figure 1:**
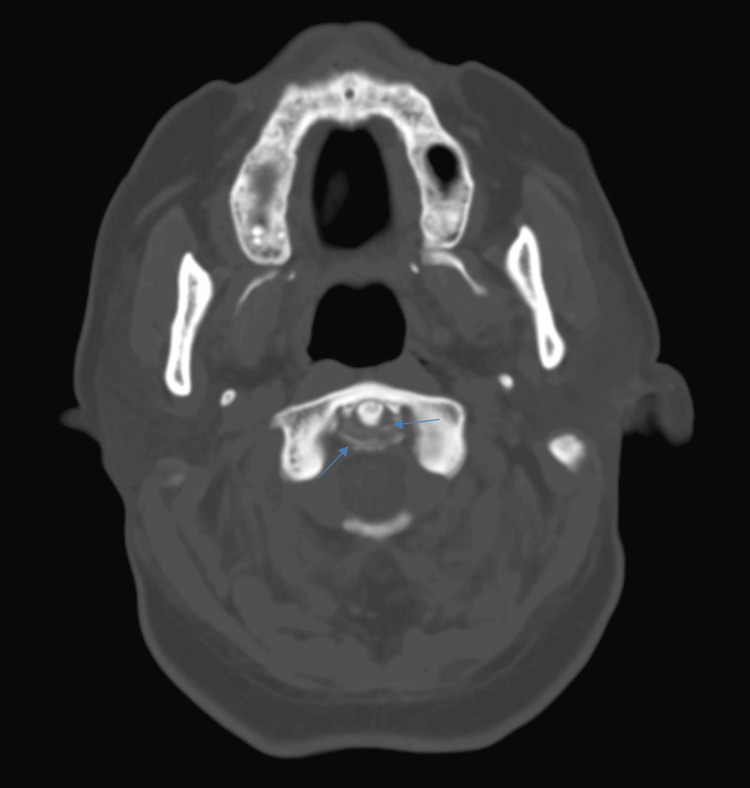
Axial CT scan of the cervical spine CT scan of the patient's cervical spine demonstrated calcifications around the odontoid process (dens) of C2, consistent with crowned dens syndrome. The calcifications are visualized encircling the dens, forming a crown-like structure in the transverse retro-odontoid ligament. This imaging finding is characteristic of calcium pyrophosphate deposition (CPPD) and correlates with the patient’s clinical presentation of neck pain, fever, and elevated inflammatory markers.

On hospital day nine, the patient began reporting increasingly severe neck and shoulder pain with stiffness. Physical examination revealed marked tenderness with neck range of motion, as well as on palpation of the paraspinal regions. Combined with recurrent fevers and elevated inflammatory markers, this raised clinical suspicion for an etiology other than meningitis. The admission CT scan of the head (Figure 1) was reviewed on hospital day 10, focusing on the cervical spine, which revealed two curved linear calcifications around the odontoid process along the region of the transverse ligament. This finding confirmed a diagnosis of CDS. Subsequent MRI demonstrated pannus formation posterior to the odontoid process, without evidence of compressive changes or cervical myelopathy. 

The patient was started on IV methylprednisolone 48 mg daily for three days, which resulted in a remarkable improvement in neck pain and upper extremity mobility. While treatment for CPPD flares includes glucocorticoids, NSAIDs, colchicine, or IL-1 inhibitors, IV methylprednisolone was chosen (rather than NSAIDs or colchicine) due to the severity of his symptoms and the proximity of sensitive structures of the spine, allowing for robust and rapid reduction of his inflammation. Rheumatology was consulted, and the patient was started on colchicine 0.6 mg BID and a prednisone taper (beginning at 50 mg per day for three days, then tapered over 18 days before stopping) with further resolution of neck pain, improved upper extremity mobility, and recovery of ambulation. Follow-up with rheumatology revealed the patient’s condition had returned to baseline, with maintenance colchicine and normal ambulation and no flare-ups. His colchicine dose was reduced to 0.6 mg daily, with a plan to increase to 0.6 mg BID if symptoms recur. Repeat inflammatory markers were ordered, but the patient was not able to access laboratory services before relocating to live with his family.

## Discussion

CDS is the characteristic manifestation of CPPD disease in the axial skeleton [1]. It is primarily caused by the deposition of CPP dihydrate crystals, or less commonly hydroxyapatite crystals, within the ligaments surrounding the odontoid process of the C2 vertebra [5]. These crystal deposits trigger an inflammatory response, resulting in the hallmark symptoms of CDS, including acute neck pain, reduced cervical mobility, fever, and elevated inflammatory markers such as CRP and ESR [1,6]. The deposition of crystals, primarily in the cruciate and transverse ligaments, is best visualized on CT imaging, which reveals calcifications around the dens forming a “crown-like” appearance, often manifesting as two parallel lines on axial CT (Figure 1). This radiologic finding is essential for differentiating CDS from other conditions that present similarly, such as meningitis or infectious spondylodiscitis [1]. The differential diagnosis also includes PMR, which shares overlapping features like acute neck pain, stiffness, and elevated inflammatory markers. However, the presence of calcifications around the dens on CT, along with the clinical features described above, is a distinguishing feature of CDS, as outlined in the 2023 classification criteria for CPPD diagnosis [7]. 

The pathophysiology of CPPD is thought to involve a defect in inorganic pyrophosphate metabolism, which is generated from the breakdown of ATP by ectonucleotide pyrophosphatase/phosphodiesterase-1 (ENPP1) [8]. As inorganic pyrophosphate concentrations increase in tissues, the complexation of pyrophosphate into crystals is enhanced. These crystal deposits preferentially accumulate in fibrocartilage (such as the meniscus), hyaline cartilage, or areas of chondroid metaplasia, as the cartilage matrix provides a favorable environment for pyrophosphate complexation [1].

Once CPP crystals are deposited, they trigger a significant inflammatory response through the NLRP3 inflammasome [8]. This inflammasome activates the release of IL-1β, a potent inflammatory cytokine, which recruits neutrophils to the site of deposition. Additionally, Toll-like receptors (TLR2 and TLR4) recognize the crystals and further amplify the immune response [1]. This cascade of events leads to acute inflammation, manifesting as the fever, neck pain, and stiffness typically seen in CDS.

In addition to the metabolic and inflammatory mechanisms, genetic factors also contribute to the development of CPPD. Gain-of-function mutations in the ANKH protein, which regulates ATP transport, lead to elevated extracellular pyrophosphate levels, promoting CPP crystal formation [1,8]. Familial cases of CPPD, especially those with early or severe presentations, are often linked to these genetic mutations. Mutations in the osteoprotegerin (TNFRSF11B) gene have also been implicated, though through different pathways [1]. These genetic associations expand our understanding of CPPD predisposition beyond environmental and metabolic factors.

Imaging plays a crucial role in diagnosing CPPD. While conventional radiography is commonly used as a first-line tool, its sensitivity for detecting CPP crystals is limited. More advanced techniques, such as CT and dual-energy CT (DECT), offer superior detection of calcifications, particularly in axial joints like the cervical spine. A CT scan showing linear deposits in the transverse ligament is considered a defining feature of CDS [1].

In this case, the patient's presentation was atypical compared to other CDS cases reported in the literature. While many patients with CDS present with acute onset of severe neck pain, limited range of motion, fever, and elevated inflammatory markers, this patient initially presented with confusion, progressive generalized weakness, and failure to thrive. Neck pain and stiffness, which are typically early and prominent symptoms of CDS, appeared later in the clinical course, complicating the discovery of symptoms and the subsequent diagnosis. This atypical progression highlights the challenges in diagnosing CDS in patients with complex and undifferentiated clinical presentations. It also underscores the importance of considering CDS in the differential diagnosis of older patients with unexplained neck pain and systemic inflammatory symptoms, especially when initial workups for other conditions are inconclusive [2]. Careful evaluation of imaging findings, particularly the distinctive calcifications around the dens, is essential for establishing a definitive diagnosis.

CDS shares several clinical features with PMR, which can complicate diagnosis. Both conditions are characterized by acute neck pain, stiffness, and elevated inflammatory markers, and they are more common in older populations [4]. However, while both conditions typically respond to glucocorticoid therapy, the response in CDS is usually rapid and dramatic. A key distinguishing feature of CDS is the presence of calcifications around the dens on CT scans, which is absent in PMR [4]. Additionally, PMR often presents with pain and stiffness that is worse in the morning and improves throughout the day, primarily affecting the proximal muscles of the shoulders and pelvic girdle. In contrast, CDS is typically associated with continuous pain and stiffness localized to the neck and suboccipital region, which does not improve as the day progresses. Notably, while there is significant overlap between the presentations of PMR and CDS, pelvic girdle pain and stiffness appear to be a distinguishing symptom between the two conditions [4].

Current management guidelines for CPPD disease focus on controlling inflammation, as no treatments are available to dissolve CPP crystals [9]. Glucocorticoids remain the first-line treatment for CDS, often providing rapid symptom relief. In cases of recurrent or chronic CPPD, low-dose colchicine and potentially disease-modifying agents such as methotrexate may be used to prevent flare-ups. However, data on the long-term use of these therapies remain limited [1]. Some studies have reported success in treating CDS with NSAID therapy [10], while others suggest that high-dose steroid therapy may lead to better resolution. Notably, the absence of steroid tapering has been identified as an independent risk factor for the recurrence of symptoms [2]. Agents targeting inflammatory pathways, such as IL-1 inhibitors, represent a potential therapeutic advance and are currently under investigation [1]. It is important to note that patients receiving short-term glucocorticoid therapy for CDS should be monitored for potential adverse effects, including hyperglycemia, gastrointestinal symptoms, myopathies, and mood disturbances-especially in older populations with comorbidities. Patients should also be regularly monitored for improvement in symptoms (neck pain, cervical mobility) and normalization of inflammatory markers. 

This case highlights the importance of thorough clinical evaluation, including consultation with rheumatology when available, and the use of appropriate imaging modalities, particularly CT scans, to identify the characteristic features of CDS. Early recognition of CDS can prevent unnecessary investigations and treatments and significantly improve patient outcomes [11]. Therefore, CDS should be considered in the differential diagnosis of older patients presenting with acute neck pain, systemic inflammatory symptoms, and elevated inflammatory markers, especially when the clinical presentation is atypical or when initial imaging and workups for other conditions are inconclusive. A systemic approach that includes a thorough physical examination and history can help reveal symptoms of CDS, such as neck pain and stiffness. Laboratory studies, including ESR and CRP, can help detect an underlying inflammatory syndrome. After clinical and laboratory assessment, considering differential diagnoses, such as PMR and meningitis, helps evaluate conditions with similar presenting symptoms. If CDS is suspected, a CT scan of the cervical region should be performed to identify calcifications in the retro-odontoid ligament, confirming the diagnosis of CDS. Once confirmed, treatment with glucocorticoids ensures timely and effective symptom management. Colchicine may then be used as maintenance therapy to prevent future flare-ups of CDS [9].

## Conclusions

CDS, though rare, should be considered in the differential diagnosis of older patients presenting with acute neck pain, systemic inflammatory symptoms, and elevated inflammatory markers. The overlap in symptoms with conditions such as PMR and meningitis highlights the importance of thorough clinical evaluation and the use of appropriate imaging modalities, particularly CT scans, to identify the characteristic calcifications around the dens. Diagnosis of CDS begins with detailed assessment and history taking, where symptoms such as neck pain, stiffness, and systemic inflammation should raise suspicion for CDS. Following clinical assessment, imaging should be obtained to confirm the presence of calcifications around the dens and exclude other conditions with similar presentations. Treatment of CDS involves glucocorticoids, which typically result in rapid symptom relief. Early recognition and treatment of CDS can significantly improve patient outcomes, prevent unnecessary diagnostic studies and interventions, and shorten hospital stays.
